# New Strategies for the Prevention and Therapy of Alzheimer’s Disease Based on Stimulation of Brain Drainage and Lymphatic Clearance

**DOI:** 10.3390/ijms27052312

**Published:** 2026-02-28

**Authors:** Oxana Semyachkina-Glushkovskaya, Vladislav Sursaev, Mikhail Poluektov, Sergey Diduk, Liubov Rychkova, Irina Madaeva, Liudmila Yakubova, Jürgen Kurths

**Affiliations:** 1 Department of Biology, Saratov State University, Astrakhanskaya 83 Str., 410012 Saratov, Russia; 2 Department of Nervous Diseases and Neurosurgery, First Moscow State Medical University (Sechenov University), Bolshaya Pirogovskaya 2, Building 4, 119435 Moscow, Russia; vladislav.sursaev@mail.ru (V.S.); polouekt@mail.ru (M.P.); 3 Department of Biotechnology, Leeners LLC, 117186 Moscow, Russia; diduk.sv@leeners.pro; 4 Research Institute of Carcinogenesis of the N.N. Blokhin National Medical Research Center of Oncology, Ministry of Health of Russia, Kashirskoe Shosse 24, 115522 Moscow, Russia; 5 Federal State Budgetary Scientific Institution Scientific Centre for Family Health and Human Reproduction Problems, 664003 Irkutsk, Russia; iphr@sbamsr.irk.ru (L.R.); nightchild@mail.ru (I.M.); 6 Department of General Medical Practice and Polyclinic Therapy, Grodno State Medical University, 230009 Grodno, Belarus; yankovliuda@yandex.by; 7 Research Institute of Intelligent Complex Systems, Fudan University, 220 Handan Rd, Yangpu District, Shanghai 200437, China; 8 Potsdam Institute for Climate Impact Research, Telegrafenberg A 31, 14473 Potsdam, Germany

**Keywords:** dementia, Alzheimer’s disease, meningeal lymphatic vessels, brain drainage and clearance, photobiomodulation, pharmacological methods, sleep, physical exercise

## Abstract

Alzheimer’s disease (AD) is a serious medical challenge, representing an incurable and insidious disease. Current treatments can slow AD progression but cannot cure it. Promising new methods for AD therapy are essential for addressing the growing number of people with dementia, especially after the COVID-19 pandemic. The review highlights pioneering approaches to AD treatment based on innovative methods for the stimulation of brain drainage and clearance, in which the meningeal lymphatic vessels (MLVs) play a key role. Clinically promising noninvasive technologies using photobiomodulation for the effective clearance of metabolites, including amyloid beta (Aβ), and for the improvement of cognitive impairment during AD progression are discussed. An interesting part of the review is its analysis of innovative methods of improving the efficacy of anti-Aβ immunotherapy by stimulating MLV growth. The review is also focused on lifestyle, including sleep and physical exercises, discussing their support for the efficient lymphatic removal of waste products from the brain. Overall, the review provides an important, informative platform to excite the interest of a wide range of readers in the development of promising and clinically significant strategies for the treatment of AD, based on new strategies for the stimulation of brain drainage and clearance.

## 1. Introduction

Alzheimer’s disease (AD) is an insidious neurodegenerative disease affecting memory, cognitive functions, and behavior. AD is widely described as an epidemic of the 21st century and a global health crisis due to the progressive increase in the number of people with AD, the lack of effective therapy, and its ranking as the sixth-leading cause of death [1,2,3]. The statistics show that in 2019, there were 55 million people worldwide with dementia [2]. It has recently been suggested that, unless an effective treatment is found, this number will increase 2.5-fold to 139 million by 2050 due to an aging global population [2]. However, the first statistics from the COVID-19 pandemic suggest that the number of people with dementia may rise much higher [4,5,6,7,8,9,10,11,12,13,14]. It has been established that the SARS-CoV-2 virus causes long-term impairments to cognitive function and memory in 30% of cases, even after infection [15]. It is important to note that in the period of 2020–2021, mortality from AD increased by 145%, which is believed to be related to the COVID-19 pandemic [4].

The recognition by the scientific community of the presence of meningeal lymphatic vessels (MLVs) in the central nervous system (CNS) has opened new perspectives for the development of promising preventive and therapeutic strategies for AD [16,17,18,19,20,21,22]. MLVs are involved in brain drainage and thereby are important for the lymphatic mechanism underlying the removal of waste products from the CNS through cerebrospinal fluid (CSF) flow [16,17,23]. Therefore, MLVs are a promising target for the treatment of brain disorders associated with the extensive accumulation of metabolites and toxins, including aging, stress, sleep deficit, and AD [24,25,26,27,28]. It is suggested that the development of methods of stimulating MLV functions will facilitate progress in the treatment of various forms of dementia, including AD [23,28,29,30,31].

This review highlights the current research trends in the development of pharmacological as well as non-pharmacological approaches, including non-invasive photobiomodulation (PBM) and physical exercise, in activating MLVs, brain drainage, and clearance for the prevention and treatment of AD.

## 2. Meningeal Lymphatic Vessels as a Promising Target for the Prevention and Treatment of AD

**Figure 1:**
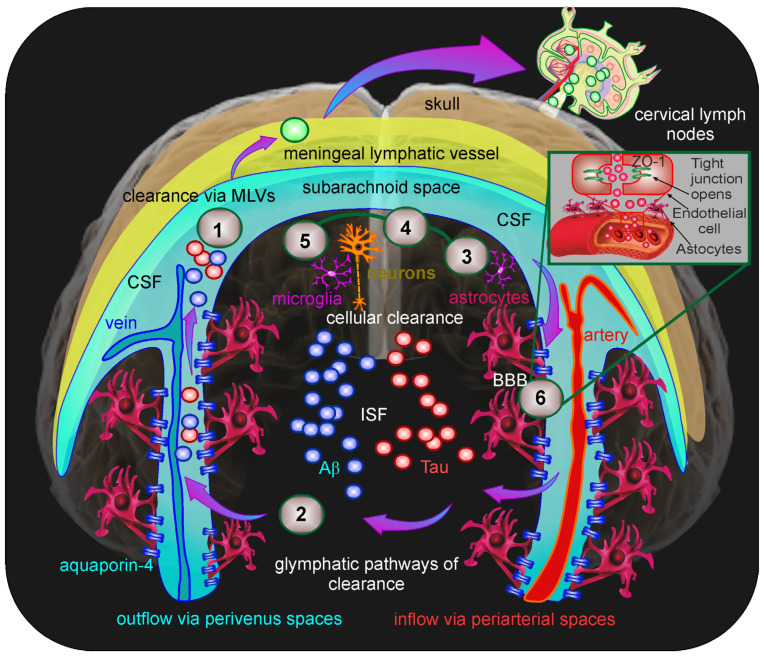
Schematic illustration of pathways for the removal of metabolites, such as Aβ and tau, from the brain: (1) Clearance of Aβ and tau through MLVs [28,65]. Since 80% of ISF is thought to be eliminated via the lymphatic route [96], the pathway of elimination of Aβ and tau via MLVs may be the major one; (2) The glymphatic pathway is based on the transfer of metabolites with the flow of ISF through glia and aquaporin-4 channels from the perivascular spaces of arteries to those of veins [98,99]; (3–4–5) Cellular mechanisms of Aβ elimination, including phagocytosis of microglia and its intracellular neutralization by neurons and astrocytes [46,47,48,49,50,51,52,53]; (6) The clearance of Aβ via transcytosis in the BBB components by the specialized endothelial transporters [54,55]; Aβ—Amyloid beta, BBB—Blood-brain barrier, CSF—Cerebral spinal fluid, ISF—Interstitial fluid, MLVs—Meningeal lymphatic vessels, Tau—Tau protein, ZO-1—Zonula Occludens-1.

Excessive accumulation of the toxic neuronal metabolite amyloid beta (Aβ) and tau protein in the brain is one of the processes that accompany the development and progression of AD [32,33,34,35,36,37]. Despite the fact that not all forms of cognitive impairment in patients with AD are accompanied by increased Aβ deposition in the brain, new strategies in the pharmacological treatment of AD continue to focus on reducing Aβ levels in the brain [35,37,38]. It is interesting to note that Aβ was first discovered in the meninges of patients with AD [39]. Aβ is a product of the vital activity of neurons, which play a crucial role in the regulation of brain homeostasis [40,41]; it is formed daily and quite intensively (one molecule per second in each neuron) [42]. This means that there must be mechanisms for the effective removal of Aβ from the brain [43,44]. Indeed, it has been established that approximately 8% of the dissolved form of Aβ is removed from the brain per hour [45]. There are various mechanisms of clearance of Aβ from the brain (Figure 1). After production, Aβ is secreted into the extracellular space and undergoes enzymatic degradation [46,47,48] or internalization via micropinocytosis [49]. Microglia also destroy Aβ by phagocytosis, although neurons and astrocytes can neutralize Aβ [50,51,52,53] (Figure 1, pathways 3–5). The clearance of Aβ via transcytosis and the blood–brain barrier (BBB) through specialized endothelial transporters, including the LDL receptor-related protein-1, is the key mechanism underlying the removal of Aβ from the brain into the bloodstream [54,55] (Figure 1, pathway 6).

It has been shown that factors, such as age, sleep deficit, stress, the SARS-CoV-2 virus, and other factors, may lead to an imbalance between the formation and clearance of Aβ, contributing to the development and progression of AD [4,5,6,7,8,9,10,11,12,13,14,56,57,58,59,60,61,62]. Excessive accumulation of Aβ triggers tau aggregation in neurons [63]. Thus, impaired Aβ clearance leads to increased levels of toxic metabolites, both inside and outside the neurons.

However, very little is known about how Aβ is eliminated from the brain via the lymphatic pathways. As noted above, Aβ was first detected in homogenates of the meninges of patients with AD [39]. In 2018, Da Mesquita et al. presented results on mouse 5xFAD, indicating the important role of MLVs in the elimination of Aβ [28] (Figure 1, pathway 1). This work clearly demonstrated that destruction of MLVs by photoablation leads to impaired lymphatic elimination of Aβ and its excessive deposition in the hippocampus and cerebral cortex, which was accompanied by a deterioration in the cognitive abilities of mice [28]. However, in immunohistological studies of the meninges of the brains of people with AD using confocal microscopy, Aβ was not detected directly in the lumens of MLVs [64]. The authors conclude that this may be due to the limitations of the confocal microscopy method and the small size of the studied area of interest. In another study on mice, Aβ was detected in the lumens of MLVs [65]. However, even if MLVs play the role of “tunnels” in the clearance of Aβ, it remains unclear how Aβ gets into MLVs from the brain tissue, including deep parts, for example, the hippocampus, since the meninges are not a part of the brain tissue.

The cerebral lymphatic vessels (CLVs) could be an obvious bridge connecting the lymphatic system and brain tissue. However, convincing evidence for this has yet to be found. Nevertheless, more and more evidence is being published suggesting that this may prove true. In 1979, Prineas was the first to discover lymphatic capillaries with immune cells in the lumens of the lymphatic vessels in human brains with various neurodegenerative diseases [66]. These results were not widely accepted because they were performed without the use of specific markers for the lymphatic endothelium and on inflamed brains. It is known that tertiary lymphatic structures can appear in the CNS during inflammation [67]. Later in 2023, Mezey et al. described lymphatic structures in healthy human brain using lymphatic endothelial markers, such as the lymphatic vessel endothelial hyaluronan receptor 1, podoplanin [68]. Authors also used Quantitative Polymerase Chain Reaction analysis and found other lymphatic endothelial markers expressed in brain tissue, including prospero homeobox protein 1 and vascular endothelial growth factor receptor 3 [68]. Despite these encouraging results, the authors did not find CLVs with distinct walls and valves. This was achieved by other researchers [69]. However, due to the limitations of using human brain biopsies, their group is still searching for additional and reproducible evidence of the presence of CLVs in the human brain. Note that there are no ideal markers of lymphatic endothelial cells in the human brain. Furthermore, human brain samples cannot be taken immediately, unlike those of animals, due to rules surrounding the certification of brain death, which requires a minimum of 2–3 h for confirmation [70]. However, studies in both humans and experimental animals have shown that oxygen stores, global electrical activity, glucose stores in the adenosine triphosphate (ATP), and expression of many proteins in the brain are lost within minutes of interrupted blood flow [71,72]. This explains why the lymphatic vessels have not yet been found in the human brain. The lymphatic vessels are transparent and, unlike the blood vessels, are not always filled with lymph, only when drainage is activated. Furthermore, the brain’s lymphatic system likely has a sparsely branched network, which, along with the need to use a fresh brain, significantly hinders progress in detecting CLVs.

There are other outstanding studies on mice, in which the authors use the term “brain lymphatic vessels” and demonstrate results with the expression of key lymphatic endothelial markers [73]. However, the authors show the brain lymphatic vessels descending from top to bottom, which could be an error in their interpretation of the data, since the meninges are quite deep in the brain tissue and are rich in the region of the middle meningeal artery, as was recently proven in the work of Albayram et al. [74].

Thus, a new player in neuroscience may soon emerge in the form of CLVs, logically resolving the main mechanisms of drainage and clearance.

It is worth noting that the discovery of MLVs has been a two-century-long story. This is due to dogmatic notions in science and the lack of reliable methods for studying the transparent lymphatic vessels. The young Italian anatomist Paulo Mascagni was the first in the world to describe MLVs, and also about 50% of all human lymphatic vessels [75,76,77,78]. This new understanding of the lymphatic system was a true scientific breakthrough, and his work became a brilliant masterpiece of anatomy. Mascagni was called the Prince of Anatomy. In the late 18th century, the Austrian Emperor Joseph II, while visiting Florence, where Mascagni worked, commissioned wax figures to be housed in the Medical Museum in Vienna, where 28 models of lymphatic drainage of tissues, including the brain meninges, are still on display [79]. Among hundreds of brains, Mascagni was able to detect MLVs filled with mercury in only some meninges [76]. Based on these studies, he created wax figurines of MLVs and the peripheral lymphatic vessels, which are still housed in the Medical Museum Vienna [79]. Despite this breakthrough discovery, it remained unrecognized due to the dogmatic thinking of other authoritative scientists, who believed that MLVs could not exist in the brain and that only Mascagni could see them [79]. In 1953, another Italian anatomist, Lecco, also identified MLVs in 4 of 30 human brain meninges [80]. However, only in 2015 was Mascagni’s discovery widely recognized by the scientific community [16,17].

It is important to note that, in the story of the discovery of MLVs, many names around the world have been underrecognized. Erickson P.P. calls this phenomenon historical amnesia [81]. Indeed, Földi scientists published a large series of papers on the study of MLVs [82,83,84,85,86]. They also published a textbook discussing the neglect of previously published research in the field of MLVs [87]. At the end of the 18th century, William Cruikshank in London (15) also studied MLVs, which he called “absorbents” [88]. Cruikshank described MLVs as absorbents on the surface of the brain, between the arachnoid and pia mater. However, Dutch scientist Ruysch was the first who observed this and called them vasa pseudo-lymphatica [89]. Ruysch was also the first to describe lymphatic valves and the direction of lymphatic flow [89]. The studies of Weed mention lymphatic drainage of CSF, but he does not credit this to MLVs [90,91]. He thought that rout for this drainage is around the cranial nerves, particularly the olfactory branches [91].

Nevertheless, for two centuries, active research has been conducted demonstrating the processes of drainage and active clearance in the brain [92,93]. Weller created the hypothesis that waste products are removed from the brain via CSF flow along perivascular spaces [94]. Later, Morris et al. experimentally confirmed the idea of Aβ removal from brain tissue through structures along the basement membrane of the cerebral blood vessels [95]. Ma showed that 80% of interstitial fluid (ISF) enters the lymphatic system [96]. The different experimental data suggest that high-molecular compounds and cells are removed from the CNS into the cervical lymphatics very quickly [93,94]. Such a process is possible only in the presence of special structures that provide direction for the movement of molecules and cells. It should be noted that in 1998, Chikly proposed, in his review, hypotheses about lymphatic structures in the human brain that could transport waste products, immune cells, and proteins [97]. Thus, the hypothesis of the possible existence of CLVs is based on a number of experimental and clinical facts indicating targeted fluid flows in the CNS that ensure brain drainage and the clearance of metabolites, waste products, and cells.

Since there is no reliable evidence for CLVs yet, the glymphatic hypothesis has emerged to fill the knowledge gap. The glymphatic hypothesis is based on the study of the possible movement of metabolites through the glia and aquaporin-4 channels with the flow of ISF in the direction from the perivascular spaces of the cerebral arteries to those of the veins [98,99] (Figure 1, pathway 2). The term “glymphatic system” means that glia perform the functions of CLVs. However, this hypothesis has been criticized, and doubts have been cast on the leading role of aquaporin-4 channels in driving the movement of brain fluids and the removal of waste products, including Aβ [92,93,100,101,102]. According to this hypothesis, it is impossible to explain how cells and large-molecular compounds, such as proteins, are rapidly cleared from the CNS. Importantly, the movement of tracers and Aβ directly through the glia was not detected. The glymphatic concept is based on the hypothesis of a possible such pathway, which remains to be proven. Nevertheless, the glymphatic hypothesis has contributed better understanding of the mechanisms responsible for the movement of dissolved compounds in ISF in the brain.

Among the various pathways for the clearance of metabolites from the brain, MLVs play a special role. Since 80% of ISF is thought to be eliminated via the lymphatic route [96], it can be hypothesized that the excretion of waste products and toxins via MLVs may be the major route of its clearance (Figure 1, pathway 1). The following facts support this hypothesis. Many studies have shown that MLV damage is accompanied by a reduced removal of various substances from the brain, including Aβ [28,103,104,105,106,107]; blood products, in the case of intraventricular [108], subarachnoid [109] or subdural hemorrhages [110]; and brain fluids after traumatic brain injury [111] or during the progression of brain cancer [112,113].

Today, the study of the role of MLVs in maintaining homeostasis and the development of brain diseases is a rapidly growing and promising area in neuroscience [18,19,20,23,28,103,104,105,106,107,108,109,110,111,112,113,114,115,116,117,118,119,120,121,122,123]. In the context of neurodegenerative diseases, the investigation of age-related changes in the function of MLVs is of particular interest [27,28,103,104,105,124,125,126,127,128]. There is evidence that a decline in the MLVs’ functions and their morphological changes with age are associated with excess Aβ deposition in the brain and cognitive dysfunction [103,104,105,125,126,129,130,131,132].

## 3. New Pharmacological Strategies for Therapy of AD via Augmentation of Brain Drainage and Clearance

Passive anti-Aβ immunotherapy is based on the use of antibodies, like aducanumab, lecanemab, and donanemab, which are approved by the US Food and Drug Administration (FDA), and aimed at reducing Aβ plaque burden and improving cognitive deficits [133,134,135,136,137,138,139,140,141,142,143,144,145,146,147]. However, despite the fact that this therapy can reduce the Aβ level in the brain and improve cognitive decline, it does not halt AD progression [136,140,142]. The clinical impact of these antibodies is limited by their negative consequences, including cerebral edema and microhemorrhages, as well as by low penetration of antibodies across the blood–brain barrier (BBB) [136,140,142]. Indeed, the data from phase 1 clinical trials using aducanumab, sponsored by Biogen, were published in Nature in 2016 and demonstrated a reduction in cognitive decline [138]. However, in 2019, Biogen (Cambridge, MA, USA) published the results of phase 3 clinical trials (1600 patients with AD participated in each, EMERGE and ENGAGE) with the monoclonal antibody aducanumab and reported microhemorrhage (3.9%, 13 of 334), edema (35.2%, 362 of 1029), headache (48 of 104, 46.6%), confusion (15 of 103, 14.6%), dizziness (11 of 103, 10.7%), and nausea (8 of 103, 8.3%) in participants [141,142]. The situation is similar with lecanemab, for which efficacy and safety remain controversia [143,144,145,146,147]. For this antibody, adverse events, including infusion-related reactions, edema, intracranial hemorrhages, and even death, have been reported [144,147].

**Figure 2:**
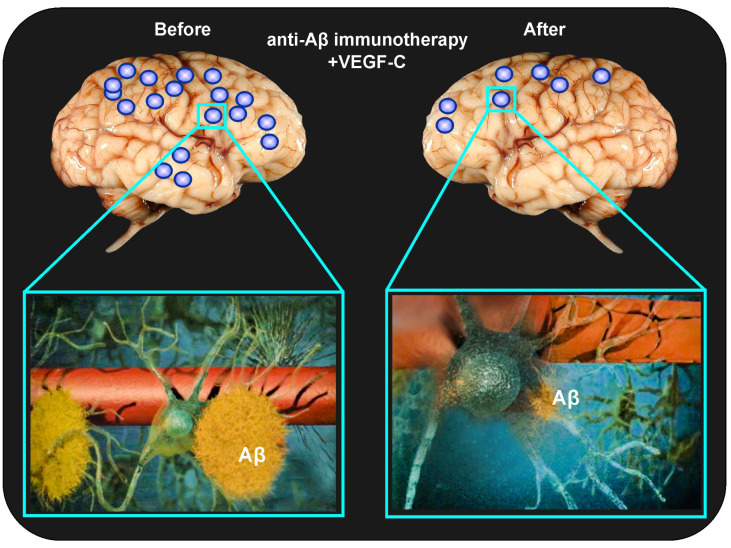
Illustration of the therapeutic effects of combined anti-Aβ immunotherapy with the introduction of VEGF-C, leading to effective lymphatic clearance of excess levels of dissolved Aβ from the brain: Aβ—amyloid beta, VEGF-C—Vascular endothelial growth factor C.

Despite the failure of clinical trials, passive anti-Aβ immunotherapy for AD has recently gained new, promising avenues thanks to a proposed strategy of combining it with the pharmacological activation of MLV functions [21,37] (Figure 2). Indeed, Da Mesquita et al. proposed a combined method of anti-Aβ immunotherapy with intrathecal administration of the vascular endothelial growth factor C (VEGF-C) for activation of lymphangiogenesis [37]. The authors clearly demonstrate that impaired MLV functions in 5xFAD male mice with AD influence the inflammatory response of the microglia, and that the stimulation of the growth of new MLVs significantly improves Aβ clearance, which can contribute to the improvement of anti-Aβ immunotherapy and better clinical outcomes [37]. It is interesting to note that the use of combined immunotherapy with VEGF-C for the treatment of glioblastoma has also been shown to be promising [112,113].

However, the meningeal lymphatic network has no anatomical connection with the blood vessels, meaning that VEGF-C cannot be used to stimulate MLVs through oral administration or by intravenous/intramuscular injection. This explains why VEGF-C was administered through the cisterna magna in Da Mesquita’s study [37]. Obviously, this method of treatment with VEGF-C is highly invasive, which significantly limits its widespread use in everyday clinical practice. However, it is possible that clinically promising methods for temporarily and safely increasing BBB permeability, such as loud music, focal ultrasound, transcranial laser treatment, or intranasal delivery, combined with lymphatic photostimulation, can improve the passage of VEGF-C into the brain tissue [148,149,150,151,152,153,154,155].

Other promising pharmacological approaches include the stimulation of aquaporin-4 channel functions. Since the aquaporin-4 channels are involved in the regulation of brain drainage, new approaches to AD therapy based on stimulation of their expression using adeno-associated virus have recently been proposed [156,157]. Indeed, it has been established in transgenic mice that the absence of aquaporin-4 channels leads to excessive accumulation of Aβ and the suppression of its lymphatic removal from the brain, while increasing their expression significantly improves the removal of Aβ from the CNS, which in turn contributes to improved memory and cognitive function [156,157].

Despite the fact that pharmacological therapy is a cornerstone of medicine, progress in the development of new drugs for the treatment of brain diseases is very slow due to the difficulties in overcoming the BBB [158,159].

Note that the creation of new drugs is a long, expensive (over $1–2 billion for each new medication), and high-risk process that takes approximately 10–15 years [160,161]. Furthermore, 90% of clinical trials of new drugs fail [161]. Therefore, the development of promising non-pharmacological approaches for the treatment of brain diseases, including AD, is a high priority in medicine.

## 4. Promising Non-Pharmacological Approaches for Therapy of AD Based on Stimulation of Brain Drainage and Clearance

**Figure 3:**
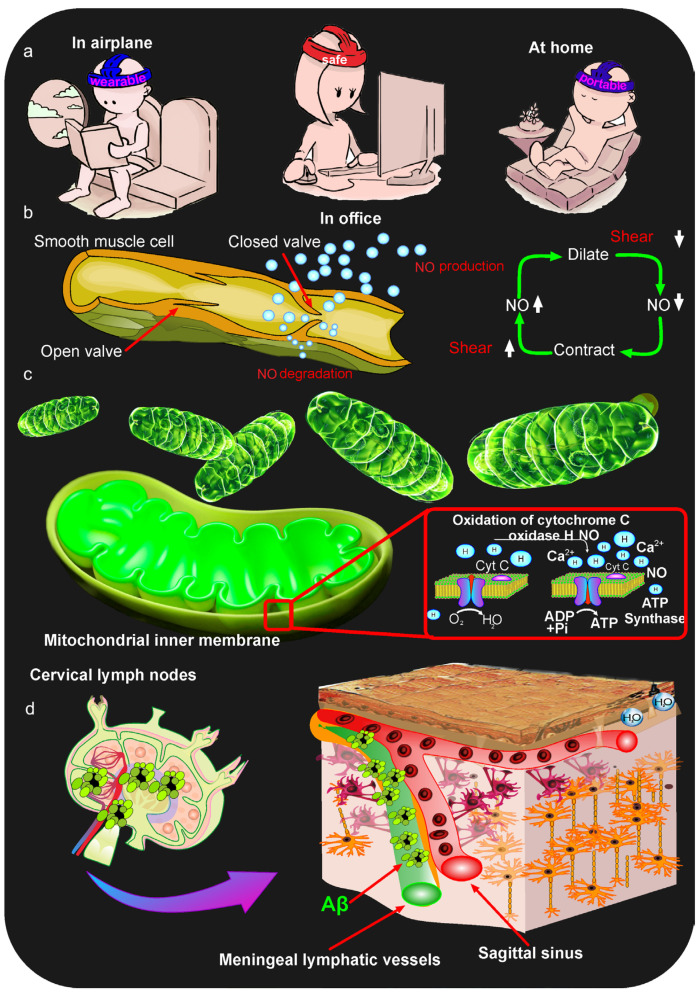
The mechanisms of PBM effects on brain drainage and clearance (**a**) Schematic illustration of a pilot portable medical device for stimulation of MLVs for AD treatment and the possibility of its use at home, in the office, on an airplane (The number of registration of clinical trial is 17491 from 27 February 2025, https://roszdravnadzor.gov.ru, accessed on 21 January 2026); (**b**) Mechanisms of PBM-mediated activation of lymphatic drainage and clearance of the brain based on photostimulation of NO-ergic and Ca^2+^-dependent processes of regulation of contractility of the lymphatic vessels (explanations in the text); (**c**) Depiction of a generally accepted scientific concept about the therapeutic effects of PBM based on the activation of the mitochondrial enzyme cytochrome C oxidase by light and an increase in NO production; (**d**) A new addition to traditional scientific concepts of the PBM-effects on the brain based on PBM-related stimulation of Aβ clearance through the lymphatic pathways, including MLVs; (**b**–**d**) The diagrams of the PBM mechanisms on brain drainage and clearance shown in the figure are based on the results of animal experiments; Aβ—Amyloid beta, ADP + Pi—Adenosine diphosphate + Inorganic phosphate, ATP—Adenosine triphosphate, Ca^2+^—Calcium channels, H_2_O—Water, NO—Nitric oxide, Cyt C—Cytochrome C oxidase.

MLVs are located in the meninges directly beneath the skull, making them an ideal target for photobiomodulation (PBM). Transcranial PBM is an effective, non-invasive alternative treatment for brain disorders, including AD, Parkinson’s, TBI, and depression [29,30,108,162,163,164,165,166,167,168]. PBM utilizes light within a therapeutic window, with wavelengths ranging from 800 to 1300 nm [163,167,168]. Light passing through the skull is largely scattered and absorbed by tissue and fluids, but the portion of the energy that reaches the brain exerts a therapeutic effect on its functions. For many years, it was traditionally believed that the therapeutic effects of light are based on its activating effect on light-sensitive structures, such as the cytochrome C oxidase [163,168]. This mitochondrial enzyme, in turn, triggers the synthesis of the ATP energy molecule and the production of vasorelaxant nitric oxide (NO) [104,108,163,168,169,170]. Thus, low-intensity light exposure is capable of triggering two integral recovery mechanisms in the brain, including an increase in neuronal energy and microcirculation, along with the necessary oxygen supply for these processes (Figure 3c).

However, PBM-dependent increases in neuronal energy and microcirculation are important but nonspecific factors in the process of nerve tissue regeneration. In other words, the scientific concept of phototherapy mechanisms has long lacked an understanding of the specific target of the light. Explanations of PBM’s therapeutic effects have been limited to molecular processes, without understanding their relationship with the systemic mechanisms of neural regeneration.

Recently, the phenomenon of the transcranial stimulation of the MLV functions and brain drainage in mice using wavelengths capable of generating singlet oxygen in pulsed mode was discovered [25,26,29,30,31,65,103,104,105,108,125,171,172,173,174,175,176]. We discuss photostimulation of MLVs by singlet oxygen in detail in our previous reviews [29,30,31]. Rafailov’s research group proves in their research that light with maximum effects at wavelengths of 1267 and 1064 nm is capable of stimulating the direct generation of singlet oxygen [177,178]. Blazquez-Castro demonstrates the entire spectrum of wavelengths (1267–1065–920–810–760–690–627–580 nm) that, by decaying in this range, can stimulate the direct formation of singlet oxygen in biological tissues [179]. Considering that light is strongly scattered and absorbed when passing through the skull, the most promising wavelengths for stimulation of MLVs are 1267 nm and 1065 nm, which produce the greatest effects in terms of singlet oxygen formation. Experiments in mice show the results of pilot studies demonstrating that light with wavelengths capable of stimulating direct singlet oxygen generation also effectively stimulates MLVs [176]. Wavelengths that are incapable of stimulating direct singlet oxygen generation do not significantly affect MLVs and brain drainage [176].

Because MLVs are located under the skull, light easily reaches their network, even with energy loss, and stimulates their functions (Figure 3d). Photostimulation of MLVs is a rapidly growing and promising field, known as neurolymphophotonics [30].

The first animal studies in this area were conducted in 2020 using a 1267 nm laser [174,175,176]. This next-generation laser is capable of stimulating the production of singlet oxygen in low concentrations [175,177,178,179]. Previously, it was believed that the generation of singlet oxygen in tissues was only possible with the use of photosensitizers. However, numerous in vitro and in vivo animal studies have demonstrated that the 1267 nm laser directly stimulates singlet oxygen production, including in neurons [175,177,178,179].

A series of experiments on rodents allowed for the study in detail of the mechanisms underlying the photostimulation of MLVs and brain drainage [65,103,104,105,108,125,173,174]. In vivo studies using two-photon microscopy and optical coherence tomography revealed that PBM activates the contractility of the lymphatic vessels, which underlie increased drainage and clearance in the brain [104,108] (Figure 3b). Interestingly, local transcranial photoexposure is accompanied by a systemic response in the lymphatic vessels. Thus, PBM of MLVs in rodents leads to increased contractility of the cervical lymphatic vessels that drain CSF from the brain to the deep cervical lymph nodes, which is clearly related to the anatomical and functional relationship between MLVs and the peripheral lymphatic system [104,108].

Several animal studies have demonstrated that the increased contractility of the lymphatic vessels in response to PBM leads to the effective removal of Aβ, blood products, and a number of studied tracers [104,108]. The key mechanism of the photoactivation of lymphatic vessel contractility is the PBM-dependent increase in the NO production in the lymphatic endothelium [104,108] (Figure 3b). NO is traditionally believed to be a vasorelaxant and dilates the blood vessels [180,181,182]. However, with regard to the lymphatic vessels, the situation is different. In the lymphatic vessels, 50% of the NO synthase is synthesized in the valves [183]. This underlies the physiological significance of the regulation of lymphatic vessel contractility [183,184,185]. Indeed, NO is released predominantly in the valves and enters locally into the lumen of the lymphatic vessel, causing its dilation and the stretching of its walls. Due to its short lifespan, NO is destroyed. However, the dilation of the lymphatic vessel leads to the development of shear stress, triggering the release of calcium (Ca^2+^) from its depots, which leads to the contraction of this portion of the lymphatic vessel (Figure 3b). This is the mechanism of peristaltic contractility in the lymphatic vessels [183,184,185]. During relaxation, the valve from which NO was released is open; during contraction, it closes, preventing the backflow of lymph [183,184,185] (Figure 3b). NO also leads to an increased permeability of the local portion of the lymphatic vessel due to a temporary decrease in the expression of tight junction proteins [176]. This promotes increased tissue drainage due to the flow of ISF into the lumen of the lymphatic vessels.

Sleep has been shown to be a natural period for activating brain drainage and clearance [186,187]. Based on this phenomenon, a new approach has been proposed of using phototherapy for brain diseases during sleep to enhance the therapeutic effects of PBM [25,26,65,125,188,189,190,191,192,193]. Indeed, PBM is ineffective in aging brains presumably due to the aging of MLVs and their morphological changes [104,105]. However, applying PBM during sleep significantly increases its effectiveness in aged mice, improving their memory and cognitive abilities [125]. PBM during sleep was found to be better than during wakefulness for activating drainage and removing metabolites such as Aβ, tau, lactate, glucose, and glutamate from the mouse brain [125]. A course of PBM during sleep has been shown to promote more rapid restoration of the damaged MLV network [65].

It is discussed that systemic responses to photostimulation of MLVs in mice is associated with an improvement of memory and cognitive function leading to the activation of brain drainage, which can contribute to optimization of the neuronal microenvironment, synaptic contacts, and neurotransmitter release [30,125].

Various animal experiments have shown that PBM-mediated MLV stimulation is effective not only for the treatment of AD but also for other brain diseases, including intraventricular hemorrhages [108], metabolic or age-related brain damage [103,104,105,194], and even cancer [195,196].

PBM of MLVs is a clinically promising approach [25,26,29,30,197]. The FDA recognizes PBM technologies as safe, and they are already used in clinical practice, and even at home [25,163,167]. Technically, PBM devices can easily be made portable, using readily available and inexpensive materials, making them both modern and commercially viable [30,198]. Thus, PBM of MLVs is a highly promising approach to advancing the treatment of brain diseases, including neurodegenerative pathologies. It should be noted that all results studying the mechanisms of PBM’s stimulating effects on MLVs were obtained in animals, which requires further verification in clinical trials to confirm the therapeutic significance of PBM. Currently, a portable medical device for phototherapy of AD through PBM-mediated activation of the MLV functions is being tested in a pilot clinical trial (Figure 3a).

Another noninvasive approach to increasing brain drainage and clearance is physical exercise [199,200,201,202,203]. A Korean group developed a specialized algorithm Aladdin for the analysis of lymph flow in MLVs using magnetic resonance imaging [204]. Later, using this algorithm, they found that physical exercise on a bicycle ergometer increases the lymph flow in the glymphatic system [200]. Other similar studies have also shown that a lifestyle with sufficient sleep and physical exercise promotes the efficient functioning of the glymphatic system [201]. Similar results have been obtained in animals, indicating that physical exercise promotes an increase in the expression of aquaporin-4 channels, which underlie the mechanisms of the glymphatic system [202,203].

Manual lymphatic drainage is another promising approach for a gentle and specialized massage technique designed to reduce swelling (lymphedema) by stimulating the lymphatic system to move excess fluid from tissues into working lymphatic vessels. It uses light, rhythmic, circular motions to move skin, primarily for treating post-surgical recovery, chronic conditions, and general wellness [205,206]. However, massage may not be sufficient for MLVs due to their location beneath the skull. Therefore, acupuncture of specific areas, such as the Feng Chi (Gallbladder 20, GB 20), may be an additional and alternative method for stimulating lymphatic drainage and brain clearance [207].

Erhardt et al. demonstrates that breathing by dilute carbon dioxide, which dilates the cerebral blood vessels, could also increase CSF flow for a short period in older adults and people with Parkinson’s disease [208]. The authors suggest that this method may also stimulate the clearance of metabolites from the brain.

Sound can also stimulate brain drainage. So, brain stimulation by sound 40 Hz promotes both neural activity in multiple brain regions and activation of the influx of CSF and the efflux of ISF in the cortex of the 5xFAD mouse model of AD [209].

Recently, the deep cervical lymphatic-vein anastomosis surgery was proposed as the new direction for invasive AD treatment [210,211,212,213]. The surgical design is a shunt that reconstructs the deep cervical lymphatic system by anastomosing it with adjacent veins, thereby gradually decreasing the accumulation of metabolic proteins in the brain (such as Aβ, Tau, and α-synuclein), and improving and alleviating the symptoms of AD [210,211,212,213].

The innovative approaches for stimulation of brain drainage and clearance in detail are discussed in the following recent reviews [214,215,216].

## 5. Conclusions

Overall, MLVs are a promising target for the development of both new pharmacological and non-pharmacological treatment strategies for brain diseases associated with suppressed brain drainage and clearance. This review is limited to a discussion of new approaches for the stimulation of the MLV functions, primarily for the treatment of AD. This is because pioneering methods in this area have been proposed for the treatment of AD [28,30]. Since this is a new, promising, and rapidly growing field, approaches based on MLV stimulation will clearly be much broader, as the important role of MLV dysfunction in the development of traumatic brain injury [111], Parkinson’s disease [217], intracranial hemorrhages [108,109,110], and brain cancer [112,113] has already been established.

Impaired glucose metabolism in the brain is considered a leading mechanism in the development of AD, which is called Type 3 Diabetes [218,219,220,221]. This review discusses the pilot results on the photostimulation of MLVs to maintain brain metabolic homeostasis during elevated brain glucose levels [92]. This area of research requires special, detailed attention, given the growing number of people with brain injuries caused by diabetes [222,223,224].

An important aspect is the development of innovative wearable and portable technologies for PBM of MLVs [26,30,198]. The pilot clinical trial of photostimulation of the meninges for the treatment of AD is currently underway, using a wearable device that can be used at home, on a plane, or in the office (The registration number is 17491 from 27 February 2025, https://roszdravnadzor.gov.ru, accessed on 21 January 2026) (Figure 3a).

Of interest is the discussion of breakthrough photo-technologies for non-invasive stimulation of the MLV’s functions, including the choice of light source, wavelength, intensity of exposure, advantages and disadvantages (for example, its ineffectiveness in elderly patients over 75 years of age [125,225,226,227]) of this method, as well as its charging devices [26,30,198].

The topic of overcoming the BBB for VEGF-C in order to stimulate lymphangiogenesis to improve anti-Aβ immunotherapy of AD requires attention. The following reviews present modern approaches to drug delivery to the brain [228,229,230].

## Data Availability

No new data were created or analyzed in this study. Data sharing is not applicable.

## Abbreviations

The following abbreviations are used in this manuscript:

Aβ	Amyloid beta
AD	Alzheimer’s disease
ATP	Adenosine triphosphate
BBB	Blood-Brain Barrier
Ca^2+^	Calcium
ISF	Interstitial fluid
MLVs	Meningeal lymphatic vessels
NO	Nitric oxide
CLVs	Cerebral lymphatic vessels
CSF	Cerebral spinal fluid
CNS	Central nervous system
PBM	Photobiomodulation
